# Distinct alterations of gut microbiota between viral- and non-viral-related hepatocellular carcinoma

**DOI:** 10.1007/s00253-023-12845-1

**Published:** 2024-01-06

**Authors:** Thananya Jinato, Songtham Anuntakarun, Nantawat Satthawiwat, Natthaya Chuaypen, Pisit Tangkijvanich

**Affiliations:** 1 Center of Excellence in Hepatitis and Liver Cancer, Department of Biochemistry, Faculty of Medicine, Chulalongkorn University, Bangkok, 10330 Thailand; 2 Doctor of Philosophy Program in Medical Sciences, Graduate Affairs, Faculty of Medicine, Chulalongkorn University, Bangkok, Thailand

**Keywords:** Gut dysbiosis, HCC, Viral hepatitis, NAFLD, 16 s rRNA, Biomarker

## Abstract

**Abstract:**

Altered gut microbiota has been connected to hepatocellular carcinoma (HCC) occurrence and advancement. This study was conducted to identify a gut microbiota signature in differentiating between viral-related HCC (Viral-HCC) and non-hepatitis B-, non-hepatitis C-related HCC (NBNC-HCC). Fecal specimens were obtained from 16 healthy controls, 33 patients with viral-HCC (17 and 16 cases with hepatitis B virus (HBV) and hepatitis C virus (HCV) infection, respectively), and 18 patients with NBNC-HCC. Compositions of fecal microbiota were assessed by 16S rRNA sequencing. Bioinformatic analysis was performed by the DADA2 pipeline in the R program. Significantly different genera from the top 50 relative abundance were used to classify between subgroups of HCC by the Random Forest algorithm. Our data demonstrated that the HCC group had a significantly decreased alpha-diversity and changed microbial composition in comparison with healthy controls. Within the top 50 relative abundance, there were 11 genera including *Faecalibacterium*, *Agathobacter*, and *Coprococcus* that were significantly enhanced in Viral-HCC, while 5 genera such as *Bacteroides*, *Streptococcus*, *Ruminococcus gnavus group*, *Parabacteroides*, and *Erysipelatoclostridium* were enhanced in NBNC-HCC. Compared to Viral-HCC, the NBNC-HCC subgroup significantly reduced various short-chain fatty acid-producing bacteria, as well as declined fecal butyrate but elevated plasma surrogate markers of microbial translocation. Based on the machine learning algorithm, a high diagnostic accuracy to classify HCC subgroups was achieved with an area under the receiver-operating characteristic (ROC) curve (AUC) of 0.94. Collectively, these data revealed that gut dysbiosis was distinct according to etiological factors of HCC, which might play an essential role in hepatocarcinogenesis. These findings underscore the possible use of a gut microbiota signature for the diagnosis and therapeutic approaches regarding different subgroups of HCC.

**Key points:**

*• Gut dysbiosis is connected to hepatocarcinogenesis and can be used as a novel biomarker.*

*• Gut microbiota composition is significantly altered in different etiological factors of HCC.*

*• Microbiota-based signature can accurately distinguish between Viral-HCC and NBNC-HCC.*

**Supplementary information:**

The online version contains supplementary material available at 10.1007/s00253-023-12845-1.

## Introduction

Hepatocellular carcinoma (HCC) is an aggressive type of liver cancer that typically develops in individuals with chronic liver disease (CLD), particularly upon hepatitis B virus (HBV) and hepatitis C virus (HCV) infection (Singal et al. 2020). Although viral hepatitis accounts for a large proportion of HCC, there is a declining trend in its incidence due to the implementation of HBV vaccine programs and effective antiviral therapies (Singal et al. 2020). In contrast, HCC with negativity in reaction with both hepatitis B surface antigen (HBsAg) and hepatitis C antibody (anti-HCV) has recently increased worldwide. This so-called non-hepatitis B-, non-hepatitis C-related HCC (NBNC-HCC) has been mostly linked to alcoholic-related liver disease (ALD) and the rising burden of non-alcoholic fatty liver disease (NAFLD) (Kulik and El-Serag 2019). NAFLD is now regarded as a leading etiological factor of the liver cancer in several Western countries, as well as in Asian populations (Li et al. 2019). In fact, ALD and NAFLD share histopathological similarity, including simple steatosis, steatohepatitis (NASH), cirrhosis, and HCC. Overall, NBNC-HCC is frequently diagnosed at more advanced stages than viral-related HCC (Viral-HCC), and the survival time of NBNC-HCC is typically worse than that of viral-related cases (Giannini et al. 2009; Hsu et al. 2020). Moreover, a meta-analysis has indicated that immunotherapeutic modality might have less effectiveness in NBNC-HCC compared to Viral-HCC (Haber et al. 2021). Thus, the differences related to underlying etiologies might lead to distinctive clinicopathological features, overall survival, and the management of individuals regarding subgroups of HCC.

Recent data have revealed the crucial role of gut microbiota as a mechanistic factor in the development and progression of CLD via communication pathways involving the biliary and portal venous systems (Schnabl and Brenner 2014). As a consequence, altered gut microbiota and impaired intestinal permeability (leaky gut) are contributed to the pathogenesis of all stages of CLD, particularly cirrhosis and HCC (Schwabe and Greten 2020). In this context, recent reports in animal studies demonstrated that gut dysbiosis and leaky gut were closely linked to the development of HCC (Iida et al. 2021; Ma et al. 2018). Additionally, altered gut microbiota and related metabolites might display a significant effect on progressive liver disease through gut-liver axis (Schwabe and Greten 2020). Regarding clinical setting, recent data demonstrated that short-chain fatty acid (SCFA)-producing bacteria were significantly diminished, whereas lipopolysaccharide (LPS)-producing genera were enhanced in patients with HCC compared with controls (Ren et al. 2019). Moreover, gut microbiota-targeted biomarkers were proposed to be potential noninvasive approached for detecting early HCC (Ren et al. 2019). Recent studies have also indicated that gut dysbiosis and bacterial metabolites play an important role in the pathogenesis of Viral-HCC and NBNC-HCC (Liu et al. 2019; Huang et al. 2020Komiyama et al. 2021; Peng et al. 2022). Despite these data, the distinct features of the gut microbial community between Viral-HCC and NBNC-HCC remain mostly unexplored.

The objective of this study was to investigate the differences of gut microbiota diversity and composition between the Viral- and NBNC-HCC subgroups based on 16S rRNA sequencing. In parallel, we also compared the levels of fecal SCFAs, as well as plasma surrogate markers of intestinal damage and microbial translocation, between the two subgroups of HCC. The results of our findings are beneficial to identify the bacteria that might be associated with the pathogenesis of different causes of HCC. Additionally, our data might help to reveal the promised use of gut microbiota-based signature as a non-invasive method and new therapeutic approach for HCC in terms of its underlying etiological factors.

## Materials and methods

### Patients

To investigate gut microbiota composition and related parameters, fecal and blood specimens were obtained from patients with HCC, who had been treated and followed up in the King Chulalongkorn Memorial Hospital, Bangkok, from 2020 to 2022. This research followed the Helsinki Declaration and Good Clinical Practice and had been approved by the Chulalongkorn University’s Institutional Review Board (IRB no. 049/63). The diagnosis of HCC was documented by dynamic computed tomography (CT) and/or magnetic resonance imaging (MRI) regarding the clinical guideline of the American Association for the Study of Liver Diseases (AASLD) (Heimbach et al. 2018). HBV infection was diagnosed by serum hepatitis B surface antigen (HBsAg), while the diagnosis of HCV infection was confirmed by anti-HCV positivity and detectable HCV RNA. Patients who were infected with human immunodeficiency virus (HIV) were excluded. For the control group, the specimens were collected from individuals who were in general good health without any systemic or liver diseases. Baseline clinical parameters are measured and presented in Table 1. All specimens were kept at − 70 °C awaiting further analysis.

**Table 1:** Baseline data of patients with HCC

Characteristics	Patients with Viral-HCC (*n* = 33)	Patients with NBNC-HCC (*n* = 18)	*p*-value
Age (years)	59.0 ± 9.5	68.5 ± 9.9	0.002*
Gender (male)	28 (84.8)	15 (83.3)	0.591
Body mass index (kg/m^2^)	24.5 ± 4.5	25.2 ± 4.9	0.692
Hemoglobin (mg/dL)	12.7 ± 2.0	11.8 ± 1.9	0.128
White blood count (10^3^/mcL)	5.8 ± 2.2	6.1 ± 3.0	0.708
Platelet count (10^9^/L)	150.6 ± 82.1	186.0 ± 109.5	0.201
International normalized ratio (INR)	1.14 ± 0.14	1.14 ± 0.11	0.919
Total bilirubin (mg/dL)	1.0 ± 0.5	1.1 ± 0.9	0.533
Aspartate aminotransferase (IU/L)	68.1 ± 53.9	70.0 ± 74.7	0.919
Alanine aminotransferase (IU/L)	55.2 ± 32.5	42.2 ± 46.8	0.251
Alkaline phosphatase (IU/L)	130.5 ± 94.9	179.9 ± 225.3	0.274
Serum albumin (g/dL)	3.6 ± 0.6	3.6 ± 0.7	0.902
Alpha fetoprotein (ng/mL)	886.2 ± 3704.7	2782.0 ± 9088.1	0.362
Presence of cirrhosis	29 (87.9)	17 (94.4)	0.645
BCLC stage (0-A/B/C)	17(51.5)/10(30.3)/6(18.2)	8(44.4)/6(33.3)/4(22.2)	0.883
HBV viral load (log_10_ IU/mL)	3.87 ± 2.1	-	-
HCV viral load (log_10_ IU/mL)	5.30 ± 1.7	-	-

*IU* international units

*p<0.05

### Collection of fecal samples and microbial DNA extraction

Collecting feces samples was undertaken by the standard operating procedures (SOPs) based on the International Human Microbiome Standard (IHMS) protocol (Costea et al. 2017). Within 2 weeks of fecal sample collection, participants were asked to discontinue antibiotics, prebiotics, probiotic supplements, or proton pump inhibitors (PPIs). Fecal samples were stored in DNA/RNA Shield™ Fecal Collection Tubes (Zymo Research Corp., Irvine, CA, USA) at − 80 °C until fecal DNA extraction. The ZymoBIOMICS™ DNA Miniprep Kit (Zymo Research Corp., Irvine, CA, USA) was applied to extract total DNA from fecal samples as indicated in the manufacturer’s recommendation. The quality and concentration of DNA were assessed by using a DeNovix UV–Vis spectrophotometer (DeNovix, Wilmington, DE, USA), and the specimens were kept at − 20 °C for subsequent analysis.

### Sequencing 16S rRNA and conducting bioinformatics analysis

The Génome Québec Innovation Centre (Montréal, QC, Canada) created DNA libraries from the amplicon-based 16S rRNA (V3-V4 regions) obtained with the primer pair 341F‐805R (Klindworth et al. 2013). The pair-end sequencing was then assessed by an Illumina MiSeq platform (Illumina, San Diego, CA, USA). Cutadapt v.2.8 was applied to eliminate chimeric sequences and trim the primer and adapter sequences to acquire the clean data (Martin 2011). The high-quality sequences were clustered as amplicon sequence variations (ASVs) using DADA2 (v.1.26.0) (Callahan et al. 2016), and then used the SILVA v.138.1 16S rRNA gene database for annotation (Quast et al. 2012). Finally, the Phyloseq and Microbiome R packages (v.1.42.0 and v.1.20.0, respectively) were used to calculate relative abundance and alpha diversity (McMurdie and Holmes 2013). Chao1, Shannon, and Simpson indices were obtained under R command “microbiome: alpha” with default parameters. Principal coordinate analysis (PCoA) according to Bray–Curtis distances was then calculated by using a web-based application (MicrobiomeAnalyst, https://www.microbiomeanalyst.ca/) (Dhariwal et al. 2017). Additionally, the permutational multivariate analysis of variance (PERMANOVA) was performed to identify variations in the beta-diversity of gut microbial community involving different clinical conditions (Anderson 2001). Taxonomies with significant abundance across various groupings were identified by linear discriminant analysis effect size (LEfSe) (Segata et al. 2011), which were conducted in Galaxy (https://huttenhower.sph.harvard.edu/galaxy/) using LDA scores > 2 and *p* < 0.05 was applied as a cutoff value.

### Quantification of microbial metabolite genes and surrogate biomarkers

In this study, the butyryl-CoA:acetate CoA-transferase (BCoAT) gene was applied to represent the expression level of butyrate in fecal extraction. Briefly, qPCR was conducted using 4X CAPITAL™ qPCR Green Master Mix to measure the copy numbers of the *BCoAT* gene (Biotech Rabbit, Berlin, Germany), as described previously (Louis and Flint 2007). The V3-V4 regions of 16S rRNA genes were determined using primer pair 341F-785R (Klindworth et al. 2013). Both genes were identified by specific degenerated primers that had previously been documented (Klindworth et al. 2013; Louis and Flint 2007). Each sample’s cycle threshold (Ct) was evaluated to the Ct of the standard curve. Finally, the V3-V4 gene region was employed to determine and normalize the quantity of the *BCoAT* gene.

Plasma was separated from peripheral blood samples within 2 h and kept at − 80 °C for additional examination. An enzyme-linked immunosorbent assay kit (Hycult Biotech, Uden, The Netherlands) was utilized to measure lipopolysaccharide binding protein (LBP) and intestinal fatty acid binding protein (I-FABP) regarding the manufacturer’s recommendations. Additionally, the plasma samples were diluted at a ratio of 1:1000 for LBP and 1:2 for I-FABP.

### Predicting microbial community functions

Phylogenetic Investigation of Communities by Reconstruction of Unobserved States (PICRUSt2) was applied to indicate the functional profiles of microbial populations based on the marker gene (Langille et al. 2013) using the Kyoto Encyclopedia of Genes and Genomes (KEGG) Orthology (KO) database (Kanehisa et al. 2012). Statistical Analysis for Metagenomic Profile (STAMP) was employed to find differences in estimated abundance of functional profiles between groups (Parks et al. 2014). The STAMP software was employed for Welch’s *t*-test, which was adjusted for multiple testing based on the Benjamini–Hochberg false discovery rate (FDR). Results with corrected *p*-values < 0.05 were regarded as statistically significant.

### Random Forest (RF) classification

In this study, genera that exhibited significant variation in the relative abundances between the Viral-HCC and NBNC-HCC subgroups within the top 50 relative abundance were identified. These selected significant genera were considered as an important feature for a Random Forest (RF) classification, which was generated by the Python Scikit-learn (Pedregosa et al. 2011). Receiver-operating characteristic (ROC) curves were calculated by applying out-of-bag (OOB) error rates. In these curves, class zero was represented by NBNC-HCC, and class one was represented by Viral-HCC. To assess the Random Forest classifiers’ performance on our dataset, we used a 75:25 train/test split and evaluated it by examining the balance between sensitivity and specificity across all possible thresholds. We compared the results to those of a random classifier, using a threshold value of 0.5 as a baseline.

### Statistical analysis

SPSS (version 22.0.0, SPSS Inc., Chicago, IL, USA) and GraphPad Prism (version 8.0, GraphPad Software Inc., San Diego, CA, USA) were applied to analyze demographic data. One-way ANOVA and chi-square tests were applied to analyze categorical data, while Student’s *t*-tests and Mann–Whitney U tests were performed for parametric and nonparametric continuous variables, respectively. Furthermore, the Kruskal–Wallis test was conducted to verify the statistical differences in the relative abundance of microbes among the studied groups, as appropriate. Results with *p*-values < 0.05 were considered statistically significant.

## Results

### Clinical characteristics of the participants

Overall, 51 patients with HCC were enrolled, which included 33 and 18 patients in the Viral-HCC and NBNC-HCC subgroups, respectively. Regarding the Viral-HCC subgroup, there were 17 and 16 cases with HBV and HCV infection, respectively. Table 1 demonstrates the baseline data of patients in the Viral-HCC and NBNC-HCC subgroups. Comparing between them, the NBNC-HCC subgroup had a higher average age than the Viral-HCC subgroup. However, there was no difference between subgroups in terms of gender, body mass index (BMI), biochemical parameters, alpha-fetoprotein level, the presence of cirrhosis, and the severity of tumor categorized by the Barcelona Clinic Liver Cancer (BCLC) staging system (Forner et al. 2018). Additionally, 16 healthy individuals recruited as the control group were 50% male and had the average age of 32.7 ± 8.6 years.

### Gut microbial diversity and composition

**Fig. 1:**
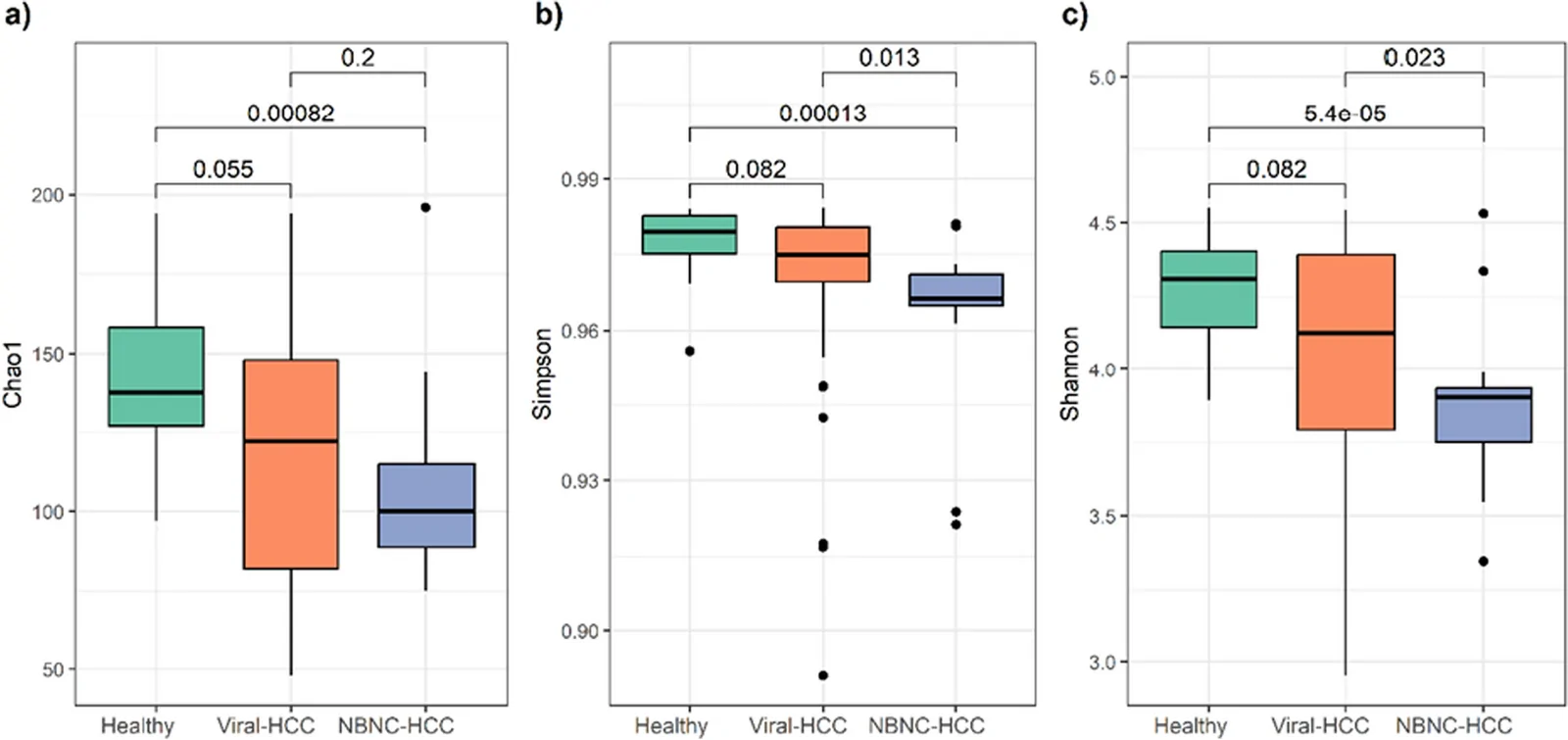
Alpha diversity indices based on **a** Chao1, **b** Simpson, and **c** Shannon compared between healthy controls, Viral-HCC, and NBNC-HCC

Alpha diversity was examined by Chao1, Simpson, and Shannon indices for the comparison among healthy controls, Viral-HCC, and NBNC-HCC (Fig. 1). Overall, the alpha diversity indices demonstrated that NBNC-HCC had a significantly lower diversity compared to healthy controls (*p* < 0.001,* p* < 0.001, *p* < 0.001, respectively). However, only Simpson and Shannon indices were shown to be significantly lower in NBNC-HCC compared to Viral-HCC (*p* = 0.013 and *p* = 0.023). In addition, the diversity based on Chao1, Simpson, and Shannon indices were lower in Viral-HCC than for healthy individuals; however, no statistical significance was observed. Furthermore, in subgroup analysis of Viral-HCC, significant difference regarding all diversity indices was not found between HBV-HCC and HCV-HCC (Supplemental Fig. S1).

**Fig. 2:**
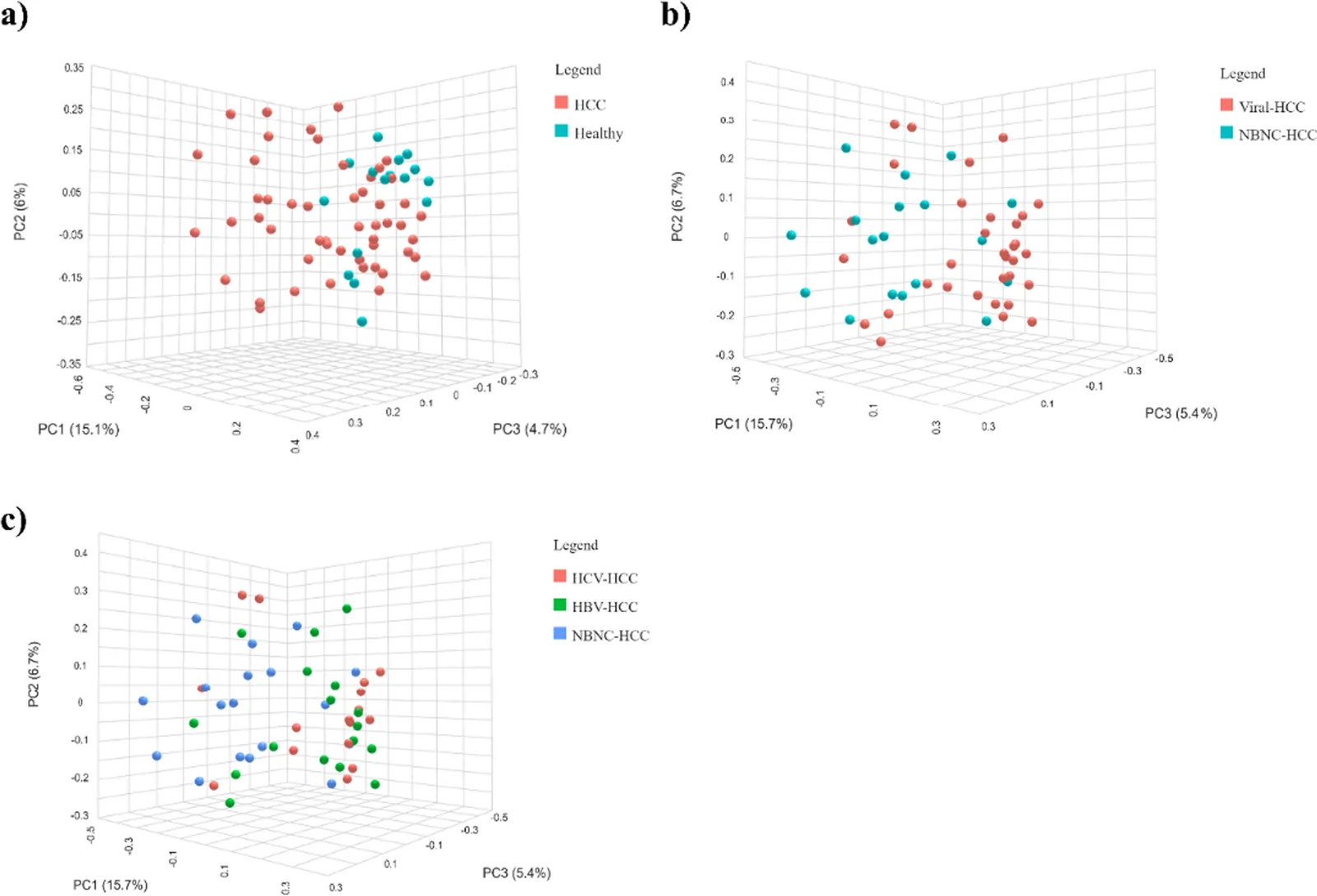
Principal coordinate analysis (PCoA) of beta diversity of gut microbiota using Bray–Curtis distance exhibit the microbial population clustering distance between **a** healthy controls vs. HCC; **b** Viral-HCC and NBNC-HCC; and **c** HCV-HCC, HBV-HCC, and NBNC-HCC

The Bray–Curtis dissimilarity and PERMANOVA tests were applied to compare the microbiota’s PCoA (Fig. 2). The results demonstrated that there were distinct differences between healthy controls and HCC (*p* = 0.006), Viral-HCC, and NBNC-HCC (*p* = 0.002), as well as between HBV-HCC vs. NBNC-HCC and HCV-HCC vs. NBNC-HCC (*p* = 0.010). However, there was no difference in the tests between HBV-HCC and HCV-HCC (*p* = 0.825).

Overall, we discovered 12 genera that showed significant differences between healthy individuals and the HCC group in the 50 genera with the highest relative abundance (Table 2). Among them, there were 5 genera enriched in HCC, including *Bacteroides*, *Streptococcus*, *Ruminococcus gnavus group*, *Veillonella*, and *Erysipelatoclostridium*. In contrast, 7 genera, including *Romboutsia*, *UCG-002*, *Lachnospiraceae NK4A-136*, *Eubacterium hallii group*, *Lachnospiraceae ND-3007 group*, *Erysipelotrichaceae UCG-003*, and *Bilophila* were underrepresented in patients with HCC.

**Table 2:** The significant taxa from the top 50 relative abundance genus level compared between healthy controls and HCC

Genera	Healthy (*n* = 16)		HCC (*n* = 51)		*p*-values
	Median	IQR	Median	IQR	
*Bacteroides*	0.039	0.082	0.095	0.096	0.042
*Streptococcus*	0.003	0.010	0.011	0.049	0.028
*Ruminococcus_gnavus_group*	0.000	0.005	0.012	0.039	0.014
*Eubacterium_hallii_group*	0.018	0.006	0.010	0.015	0.007
*Veillonella*	0.000	0.000	0.001	0.009	0.008
*UCG002*	0.008	0.019	0.002	0.013	0.034
*Lachnospiraceae NK4A136 group*	0.007	0.011	0.002	0.008	0.018
*Romboutsia*	0.005	0.006	0.000	0.007	0.014
*Lachnospiraceae ND3007 group*	0.007	0.009	0.002	0.010	0.033
*Erysipelotrichaceae UCG-003*	0.004	0.009	0.000	0.004	0.025
*Erysipelatoclostridium*	0.000	0.000	0.000	0.006	0.020
*Bilophila*	0.001	0.002	0.000	0.002	0.033

*IQR* interquartile range

Of note, significant variations of the genera between HBV-HCC and HCV-HCC were not demonstrated in our study. Therefore, we focused on the analysis of genera diversity between Viral- and NBNC-HCC. Among the genera of the top 50 relative abundance (Supplemental Fig. S2), we identified 16 genera that were significantly different between Viral- and NBNC-HCC (Mann–Whitney *U* test, *p* < 0.05). Compared to Viral-HCC, 11 genera, i.e., *Faecalibacterium*, *Agathobacter*, *Prevotella*, *Coprococcus*, *Subdoligranulum*, *Ruminococcus gauvreauii group*, *Lachnospiraceae ND3007 group*, *Erysipelotrichaceae UCG-003*, *CAG-56*, *Holdemanella*, and *Lachnospiraceae UCG-004*, were reduced in NBNC-HCC, while 5 genera, including *Bacteroides*, *Streptococcus*, *Ruminococcus gnavus group*, *Parabacteroides*, and *Erysipelatoclostridium*, were found to be enriched (Table 3).

**Table 3:** The significant taxa from the top 50 relative abundance genus level compared between Viral- and NBNC-HCC

Genera	Viral-HCC		NBNC-HCC		*p*-values
	Median	IQR	Median	IQR	
*Bacteroides*	0.085	0.099	0.123	0.076	0.029
*Faecalibacterium*	0.081	0.063	0.031	0.061	0.004
*Streptococcus*	0.005	0.027	0.026	0.086	0.035
*Agathobacter*	0.028	0.034	0.000	0.023	0.014
*Prevotella*	0.000	0.057	0.000	0.000	0.049
*Ruminococcus gnavus group*	0.002	0.023	0.028	0.038	0.001
*Coprococcus*	0.012	0.030	0.000	0.009	0.022
*Subdoligranulum*	0.017	0.029	0.000	0.000	< 0.001
*Parabacteroides*	0.003	0.006	0.010	0.021	0.034
*Ruminococcus gauvreauii group*	0.004	0.011	0.000	0.002	0.041
*Lachnospiraceae ND3007 group*	0.005	0.010	0.000	0.003	0.006
*Erysipelotrichaceae UCG-003*	0.001	0.014	0.000	0.000	0.016
*CAG56*	0.002	0.010	0.000	0.000	0.001
*Holdemanella*	0.000	0.013	0.000	0.000	0.048
*Erysipelatoclostridium*	0.000	0.001	0.005	0.013	0.001
*Lachnospiraceae UCG-004*	0.004	0.006	0.000	0.004	0.010

### Gut microbial metabolites and microbial translocation

**Fig. 3:**
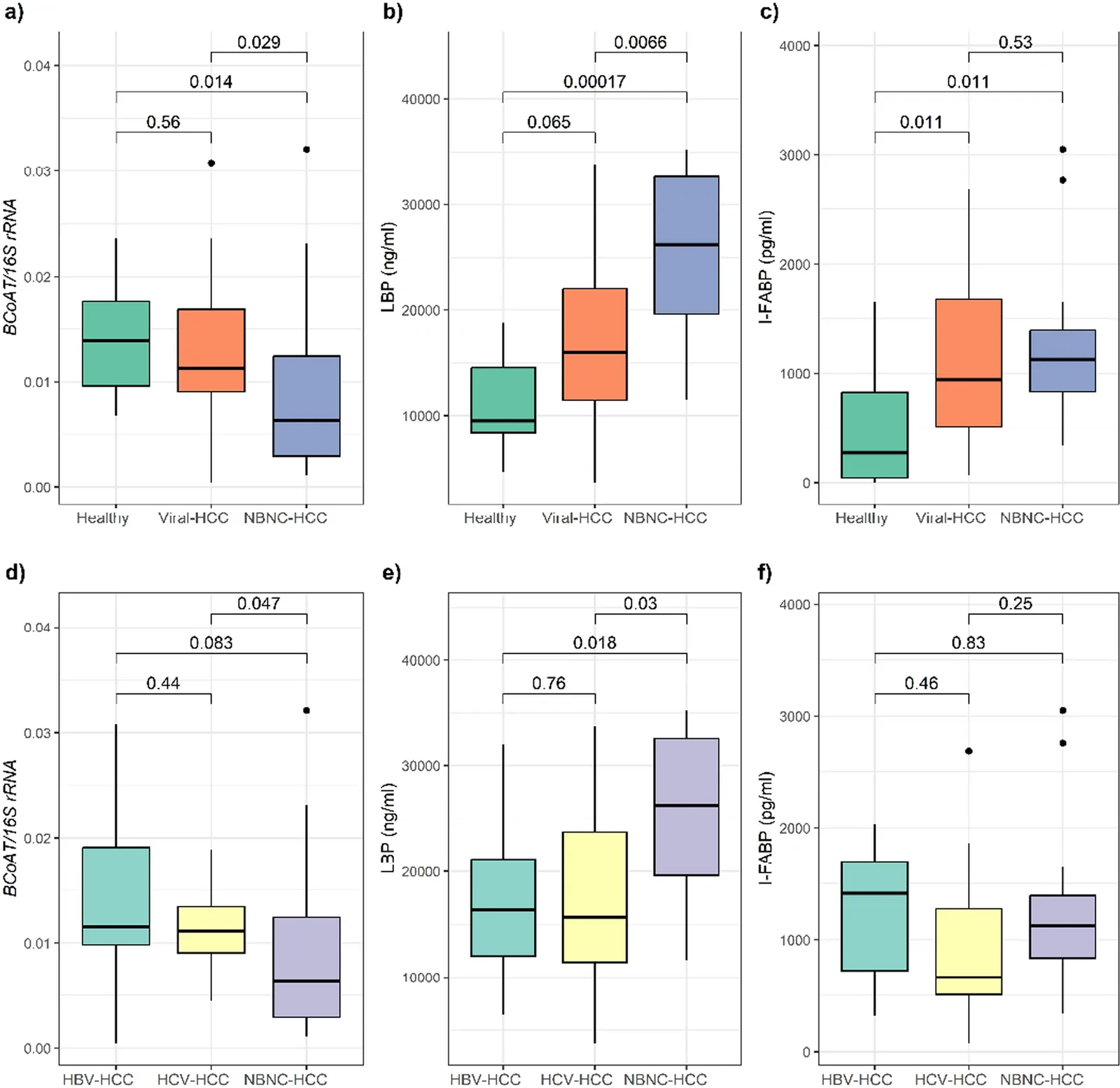
Gut microbial metabolite genes (*BCoAT*) and surrogate biomarkers (LBP and I-FABP) in healthy controls, Viral-HCC, and NBNC-HCC (**a**–**c**) and HBV-, HCV-, and NBNC-HCC (**d**–**f**)

To determine the level of fecal butyrate, the *BCoAT* gene in form of copy numbers was assessed by qPCR (Fig. 3a, d). The level of *BCoAT* copies was alleviated in NBNC-HCC compared with healthy individuals (*p* = 0.014) and Viral-HCC (*p* = 0.029). Moreover, NBNC-HCC had a lower *BCoAT* level compared with HBV-HCC and HCV-HCC, but a significant difference was observed only in HCV-HCC (*p* = 0.047).

The intestinal impairment and microbial translocation were verified by detection of plasma I-FABP and LBP, respectively. Overall, plasma LBP was significantly higher in NBNC-HCC than in healthy controls (*p* < 0.001) and Viral-HCC (*p* = 0.007) (Fig. 3b). Moreover, the I-FABP level was decreased in the control group compared with the Viral-HCC (*p* = 0.011) and NBNC-HCC subgroups (*p* = 0.011) (Fig. 3c). Regarding HCC subgroup analysis, we found that the LBP level was increased in NBNB-HCC compared to HBV-HCC (*p* = 0.018) and HCV-HCC (*p* = 0.030) (Fig. 3e) while there was no significant difference in the I-FABP level among these subgroups (Fig. 3f).

### Discriminant analysis and prediction of functional pathways in subgroups of HCC

**Fig. 4:**
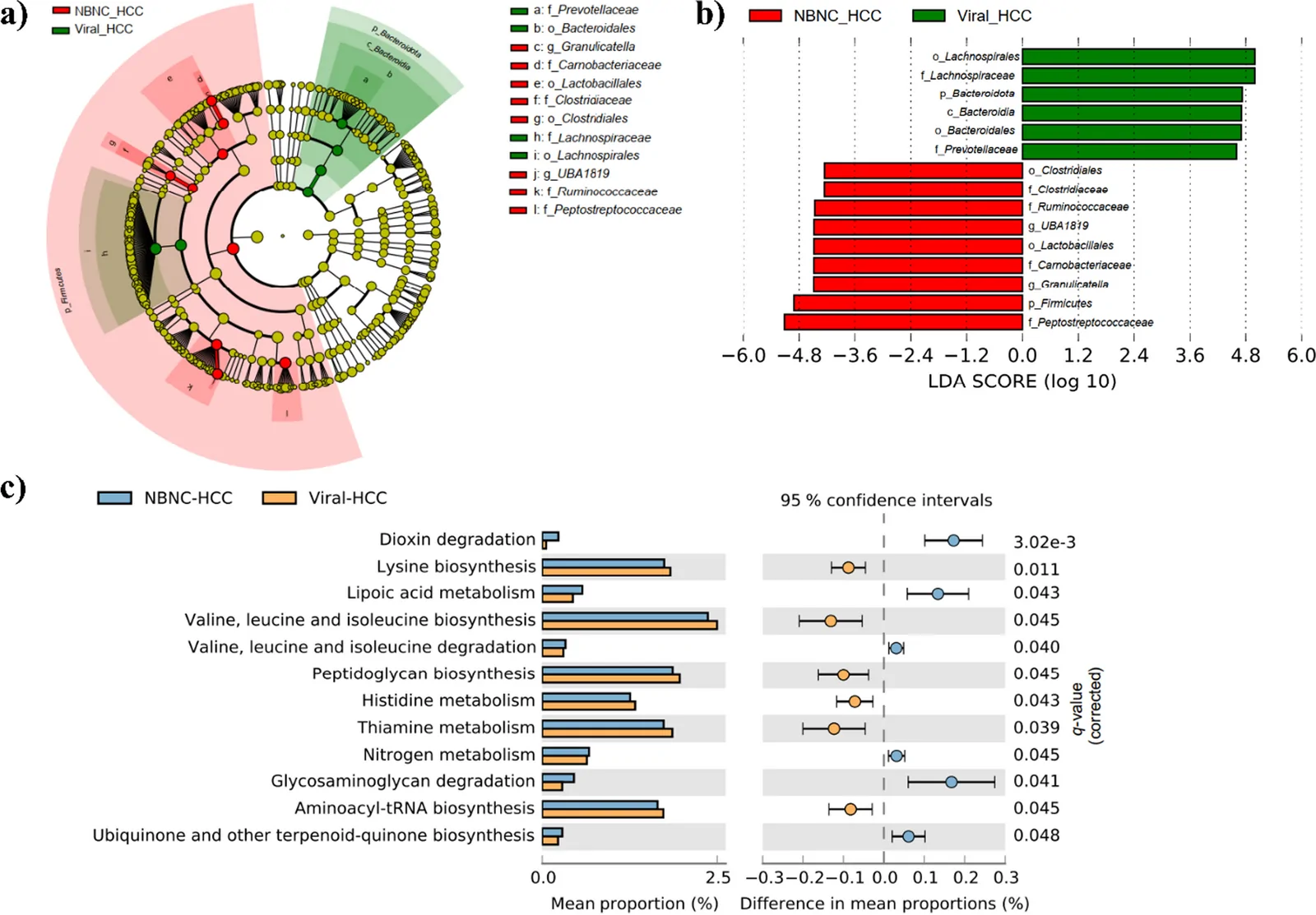
LEfSe identifies the microbiota that differed the most between NBNC-HCC and Viral-HCC (**a**, **b**). The LDA score for NBNC-HCC is negative, while the score for Viral-HCC is positive. An extended error bar plot demonstrates the difference in KEGG pathways in NBNC-HCC and Viral-HCC (**c**). The proportion on the left side refers to the potential abundance of microbes that have each functional feature, while the difference between the proportions (which represents effect sizes) is calculated for each feature

Linear discriminant analysis effect size (LEfSe) analysis was applied to assess the highest differences in microbiota compositions between Viral-HCC and NBNC-HCC (Fig. 4a, b). In Viral-HCC, we discovered a higher abundance of families *Prevotellaceae* and *Lachnospiraceae* than in group NBNC-HCC. In contrast, 4 families including *Clostridiaceae*, *Ruminococcaceae*, *Carnobacteriaceae*, and *Peptosreptococcaceae*, as well as 2 genera including *UBA1819* and *Granulicatella*, were enhanced in NBNC-HCC compared with Viral-HCC.

Functional analysis of microbial communities in subgroups of HCC was further predicted based on PICRUSt2. In total, 153 KEGG pathways were identified across all samples. Regarding the threshold of false discovery rate (FDR) < 0.05, 12 key KEGG pathways were differentially abundant between Viral-HCC and NBNC-HCC. These pathways were associated with genetic information processing, xenobiotics biodegradation, and glycan biosynthesis, as well as metabolisms of several substances including amino acids, cofactors, and vitamins. Moreover, these as significant different predicted pathways were mostly related to tumor formation and immune regulation (Fig. 4c). Among them, the most distinct functional pathway was dioxin degradation, which was found significantly more often present in NBNB-HCC compared with Viral-HCC.

### Random Forest classification

**Fig. 5:**
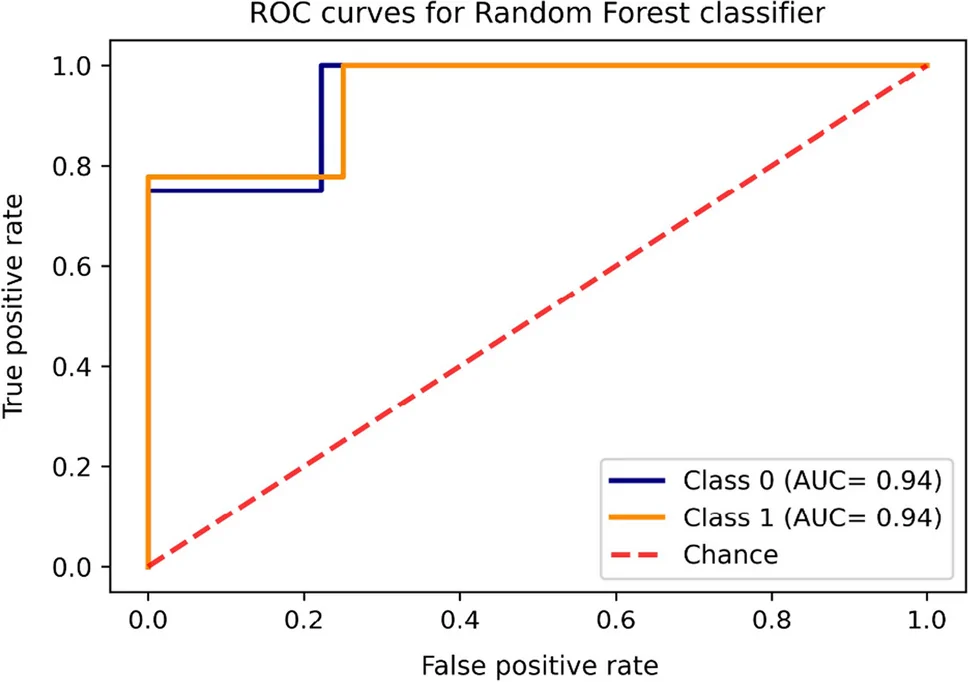
ROC curves for Random Forest classifier estimating ability to predict Viral-HCC and NBNC-HCC based on Random Forest classifier. The curve corresponds to the specificity and sensitivity in differentiating between Viral-HCC (class 1) and NBNC-HCC (class 0)

The Random Forest algorithm has been chosen to derive gut microbiota signatures in previous studies related to HCC (Huang et al. 2020; Kang et al. 2022; Piñero et al. 2019). In this study, this model was trained on a training set and tested in an additional test set. ROC curve analysis based on the Random Forest classifier was calculated using the 16 genera that significantly differed between Viral- and NBNC-HCC (Table 3). In this context, our data demonstrated that the highest diagnostic value obtained from the selected genera displayed an area under the ROC curve (AUC) of 0.94 (Fig. 5), with high sensitivity (97.0%) and specificity (83.3%).

## Discussion

Gut dysbiosis, specified as the disproportion between beneficial and pathogenic bacteria, has been linked to oncogenesis of several gastrointestinal cancers including HCC (LaCourse et al. 2021; Schnabl and Brenner 2014). Despite these reports, in-depth data regarding gut microbiota characteristics in differentiating between distinct etiological factors of HCC are limited. This report represents one of the first studies that directly compares gut dysbiosis between patients with Viral-HCC and NBNC-HCC. Our data demonstrated that 16 genera significantly differed between Viral-HCC and NBNC-HCC and such microbiota-based signature had a high diagnostic performance in distinguishing between the two subgroups of HCC. These data may help better understanding of altered bacterial communities in the development of HCC according to the underlying etiologic factors. Moreover, our findings might encourage the clinical use of microbiota-based signatures as noninvasive diagnostic markers and the possibility of therapeutic interventions by modulating gut microbial composition.

### Gut microbial diversity and composition

Gut microbiota has been indicated to play a pivotal role in several CLD, ranging from the development of chronic hepatitis and cirrhosis, as well as HCC (Schnabl and Brenner 2014). Our study demonstrated that compositional shifts of gut microbiota were considerably remarked in patients with HCC compared with healthy subjects. In particular, alpha diversity was diminished in the HCC group as indicated by Chao1, Simpson, and Shannon indices. Additionally, there were 12 genera that significantly differed between the HCC group and healthy individuals. For instance, the HCC group had increased levels of *Bacteroides*, *Streptococcus*, *Veillonella*, and *Ruminococcus* accompanied by decreased abundances of *Eubacterium hallii*, *Romboutsia*, and *Lachnospiraceae.* Previous data in both mouse models and human studies demonstrated that *Bacteroides* was significantly elevated in HCC and linked to increase in several systemic inflammatory and immunological markers (Ponziani et al. 2019; Xie et al. 2016). Moreover, levels of several opportunistic pathogenic bacteria such as *Veillonella* and *Streptococcus* were enhanced in HCC. These observations were in line with a recent Chinese report of primary liver cancers (Deng et al. 2022).

Of note, microbial diversity in the fecal samples progressively declined from the Viral-HCC to NBNC-HCC subgroups. Among Viral-HCC, however, alpha diversity and richness of gut microbiota were comparable between patients infected with HBV and HCV. As a result, we decided to combine these subgroups of Viral-HCC together and further compared them with NBNC-HCC. Interestingly, 11 genera such as *Faecalibacterium*, *Agathobacter*, *Coprococcus*, *Subdoligranulum*, and *Lachnospiraceae* were significantly enriched in the Viral-HCC subgroup, while 5 genera including *Bacteroides*, *Ruminococcus gnavus*, *Streptococcus*, *Parabacteroides*, and *Erysipelatoclostridium* were significantly increased in NBNC-HCC. These data were consistent with previous reports demonstrating that NAFLD-HCC had increased abundance of *Bacteroides*, *Ruminococcus*, and *Streptococcus*, among other genera (Behary et al. 2021; Ponziani et al. 2019). Moreover, the genera *Bacteroides* and *Ruminococcus* were shown to be associated with liver inflammation, particularly in NAFLD individuals with advanced liver disease (Boursier et al. 2016; Loomba et al. 2017). *Streptococcus* was previously found as a predominant genus in patients with advanced ALD and might be a useful biomarker for the assessment of liver damage (Zhong et al. 2021). Thus, it might indicate that the above-mentioned bacterial genera played an essential part involving NBNC-HCC development through ongoing liver inflammation and hepatocarcinogenesis.

In agreement with our data, a recent study demonstrated distinct microbial compositions between HBV-related HCC and NBNC-HCC (Liu et al. 2019). For instance, fecal specimens collected from individuals with NBNC-HCC displayed pathogenic bacteria such as *Enterococcus* and *Escherichia-Shigella* but exhibited a smaller number of anti-inflammatory bacteria including *Faecalibacterium* and *Ruminoclostridium* (Liu et al. 2019). Additionally, a recent published report demonstrated that *Faecalibacterium* was enhanced in HBV-related cirrhosis, suggesting its abundance was related to disease severity in viral hepatitis (Shen et al. 2023). *Faecalibacterium prausnitzii*, an oxygen-sensitive and SCFA-producing bacterium, plays an crucial role in promoting gut health through its anti-inflammatory and mucosal-protective properties (Lopez-Siles et al. 2017), and its reduction has been connected to several disorders including severe ALD (Sarin et al. 2019). Furthermore, a decline in *Faecalibacterium* was observed in NAFLD comparing with non-NAFLD in a study cohort with BMI- and sex-matched (Iino et al. 2019). Although the mechanism linking *Faecalibacterium* abundance to NAFLD has not been clearly elucidated, it was recently revealed in a mouse model that the combination of SCFAs and other metabolites produced from this genera might contribute to the development and severity of NAFLD (Hu et al. 2022).

### SCFA-producing bacteria and fecal BCoAT gene

SCFAs, mainly acetate, propionate, and butyrate, exert several physiological functions such as enhanced gut barrier integrity, regulated immune function and inflammatory process (Nogal et al. 2021). Moreover, animal and clinical investigations demonstrated that SCFAs exhibit antineoplastic properties through various molecular mechanisms (Jaye et al. 2022). Notable, data in mouse HCC models also showed that gut microbiota derived SCFAs might enhance the efficacy of immunotherapy for HCC (Hu et al. 2023). In our study, several SCFA-producing bacteria, including *Faecalibacterium*, *Agathobacter*, *Subdoligranulum*, *Coprococcus*, and *Lachnospiraceae* were declined in NBNC-HCC compared to Viral-HCC. Among them, *Subdoligranulum* is a strictly anaerobic, butyrate-producing bacterium that was negatively linked to several metabolic risks in patients with NAFLD (Aron-Wisnewsky et al. 2020). Compared to healthy individuals, *Subdoligranulum* was also found to be less abundant in alcoholic cirrhosis (Van Hul et al. 2020). Moreover, the relative abundance of *Faecalibacterium*, *Lachnospira*, and *Agathobacter* progressively diminished in patients with advanced ALD (Zhong et al. 2021). In this report, we also observed reduced fecal *BCoAT* gene copy numbers, a semiquantitative measurement of butyrate (Louis and Flint 2007), in NBNC-HCC compared to Viral-HCC and healthy individuals.

### Plasma levels of LBP and I-FABP

Increasing evidence has shown that gut dysbiosis influences progressive liver disease and hepatocarcinogenesis through the consequences of leaky gut and microbial translocation (Chopyk and Grakoui 2020; Xu et al. 2022). Indeed, several studies have shown that gut-derived components, including LPS, could lead to hepatocyte injury, progressive fibrosis, and HCC development (Xu et al. 2022). In ALD, chronic alcohol consumption appears to greatly contribute to bacterial overgrowth, gut inflammation, and barrier disruption (Sarin et al. 2019). In the context of NAFLD, leaky gut is also of relevance in ALD, leading to elevated gut permeability and bacterial translocation (Lang and Schnabl 2020), as recent data in animal models and patients demonstrated that gut dysbiosis and impaired gut barrier were observed to be early events in NAFLD pathogenesis (Mouries et al. 2019). In addition, elevated richness of alcohol-producing bacteria and increased circulating ethanol were shown in advanced NAFLD, suggesting that NAFLD and ALD share similar pathogenesis (Zhu et al. 2013). In our report, we further analyzed plasma levels of LBP and I-FABP, the surrogate markers of microbial translocation and intestinal damage, respectively. LBP, a 50-kD polypeptide binding to the lipid A portion of LPS, has gained increasing attention as an appropriate alternative biomarker of LPS because of its stability in the circulation (Vanuytsel et al. 2021). In this report, we demonstrated that plasma LBP was increased in NBNC-HCC compared with Viral-HCC and healthy controls. Together, our data could indicate that altered gut microbiota composition in HCC, particularly diminished SCFA-producing bacteria, together with elevated intestinal permeability and microbial translocation, were predominantly detected in NBNC-HCC.

### Clinical aspects of gut dysbiosis

Regarding clinical aspects, the natural history of NBNC-HCC is more insidious and has a poor prognosis compared with Viral-HCC (Giannini et al. 2009; Hsu et al. 2020). Moreover, a meta-analysis indicates that immunotherapies are less effective in NBNC-HCC than patients infected with viral hepatitis (Haber et al. 2021). Recent mechanistic evidence demonstrates that NBNC-HCC, particularly NAFLD-HCC, has reduced responsiveness to immunotherapies, probably due to the dysfunction of intrahepatic and peripheral immune surveillance (Behary et al. 2021; Pfister et al. 2021). Interestingly, increasing data have also revealed unique fecal microbial features in various cancers that might attribute to antitumor immunity (Davar and Zarour 2022). In advanced HCC, for example, increased abundance of *Lachnospiraceae* was linked to better progression-free survival (PFS) and overall survival (OS), while the enrichment of *Veillonella* was associated with reduced PFS and OS (Mao et al. 2021). In this regard, we found that *Lachnospiraceae ND3007 group* declined significantly in NBNC-HCC compared to Viral-HCC.

Through functional analysis of microbial communities, our results suggested that there were different functional pathways related to tumor formation and immune regulation that were driven by diverse subgroups of HCC. Among them, dioxin degradation was identified as the most distinctive pathway between Viral-HCC and NBNC-HCC. This pathway modulates the host-microbial interactions and its signaling has been documented in several CLD (Dong and Perdew 2020). In NAFLD, most studies in animal models showed that its signaling exerted deleterious effects on steatosis development and disease progression (Wang et al. 2022) and could mediate transcriptional regulation contributing to tumor development (Kerkvliet 2009). Collectively, these data suggest that gut dysbiosis and its related functional pathways modulating immune responses across diverse etiologies of HCC might play roles in therapeutic outcomes of HCC.

Study limitations and future considerations, our study might have some limitations. First, this report was conducted in a tertiary center and had a relatively small sample size, which might lead to biased estimates of gut microbiota composition in the subgroups of HCC. Second, the complex and heterogeneous compositions of gut microbiota are influenced by several factors, including dietary, lifestyle, and environmental backgrounds. In this context, our study was limited by the insufficient information of these factors that could potentially affect the interpretation of microbial profiling. Third, we could not exclude the possibility of occult HBV infections in the NBNC-HCC subgroup even with HBsAg-negativity, particularly in the endemic area of HBV.

In conclusion, our data provides essential evidence of direct comparison between the Viral- and NBNC-HCC subgroups in respect of gut microbiota and related metabolites. These findings highlight the promising use of gut microbiota signature for the diagnosis and therapeutic approaches involving subgroups of HCC. In the future, there will be a need for additional studies, which include larger sample sizes with a comprehensive assessment of dietary and environmental factors, to verify our observations regarding the distinct gut dysbiosis associated with different etiologies of HCC.

## Supplementary information

Below is the link to the electronic supplementary material.

Supplementary file1 (PDF 281 KB)

## Data Availability

The data were uploaded to the Short Read Archive of the National Center for Biotechnology Information (NCBI) with the Bioproject accession number PRJNA951258 and were available upon reasonable request to the corresponding authors.
