# Supplementary material for: Distinct alterations of gut microbiota between viral- and non-viral-related hepatocellular carcinoma
Source: Appl Microbiol Biotechnol. 2024 Jan 6;108:34. doi: 10.1007/s00253-023-12845-1 (PMC10771587; doi:10.1007/s00253-023-12845-1)
Supplement: Supplementary file 1 [file 253_2023_12845_MOESM1_ESM.pdf]

Supplemental Material

Applied Microbiology and Biotechnology

Distinct alterations of gut microbiota between viral and non-viral-related hepatocellular carcinoma

Thananya Jinato[1,2], Songtham Anuntakarun[1], Nantawat Satthawiwat[1], Natthaya Chuaypen[1]*, Pisit Tangkijvanich[1]*

[1]Center of Excellence in Hepatitis and Liver Cancer, Department of Biochemistry, Faculty of Medicine, Chulalongkorn University, Bangkok, 10330, Thailand

[2]Doctor of Philosophy Program in Medical Sciences, Graduate Affairs, Faculty of Medicine, Chulalongkorn University, Bangkok, Thailand

*Correspondence:

Pisit Tangkijvanich, M.D., Center of Excellence in Hepatitis and Liver Cancer, Faculty of medicine, Chulalongkorn University, Bangkok, 10330, Thailand.

pisittkvn@yahoo.com; Tel: +66 2 256 4482

*Co-correspondence:

Natthaya Chuaypen, PhD. Center of Excellence in Hepatitis and Liver Cancer, Faculty of medicine, Chulalongkorn University, Bangkok, 10330, Thailand.

natthaya.c@chula.ac.th; natthaya.ch56@gmail.com; Tel: + 66 2 256 4482

Supplemental Material

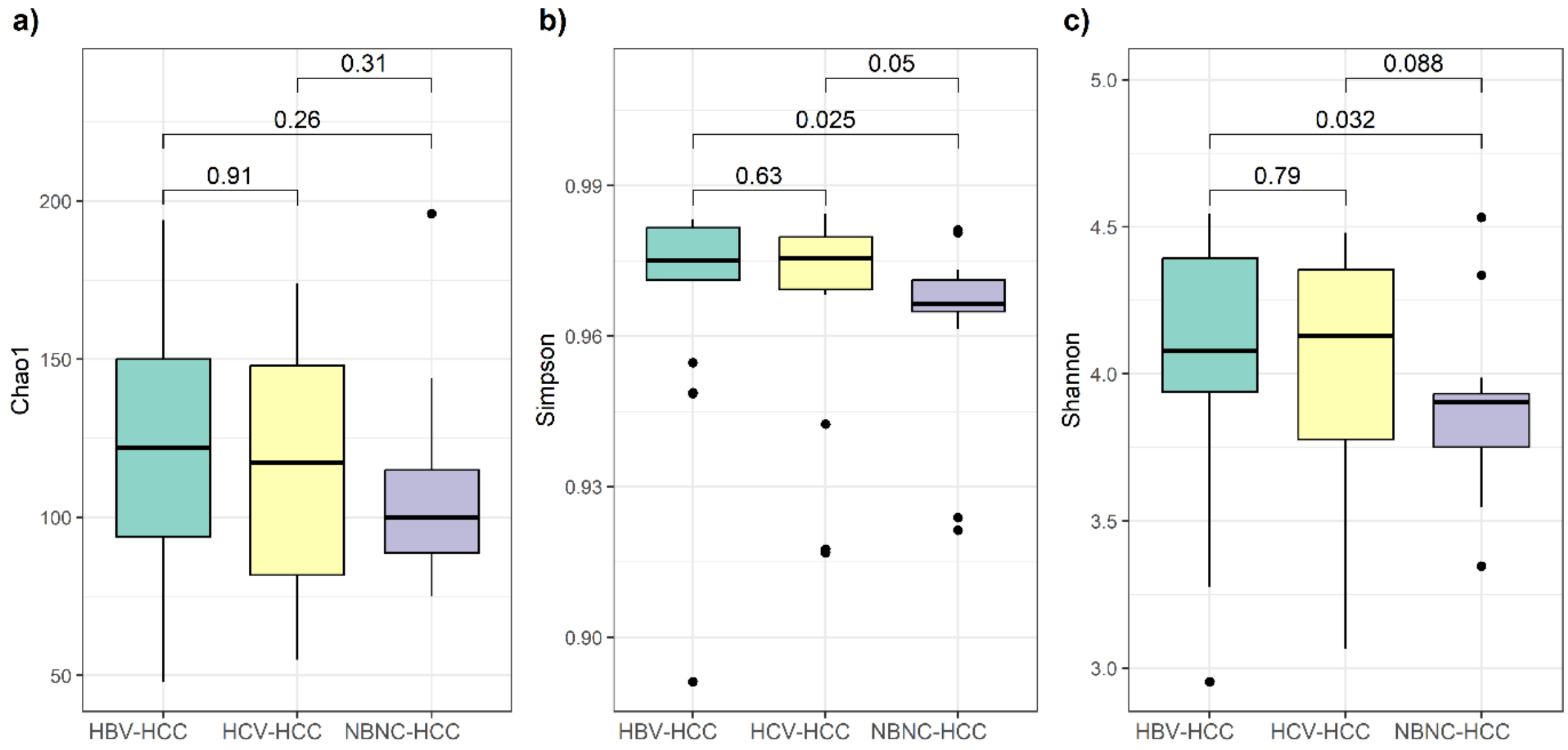

Fig. S1 Alpha diversity indices based on (a) Chao1, (b) Simpson, and (c) Shannon compared between HBV-HCC, HCV-HCC, and NBNC-HCC

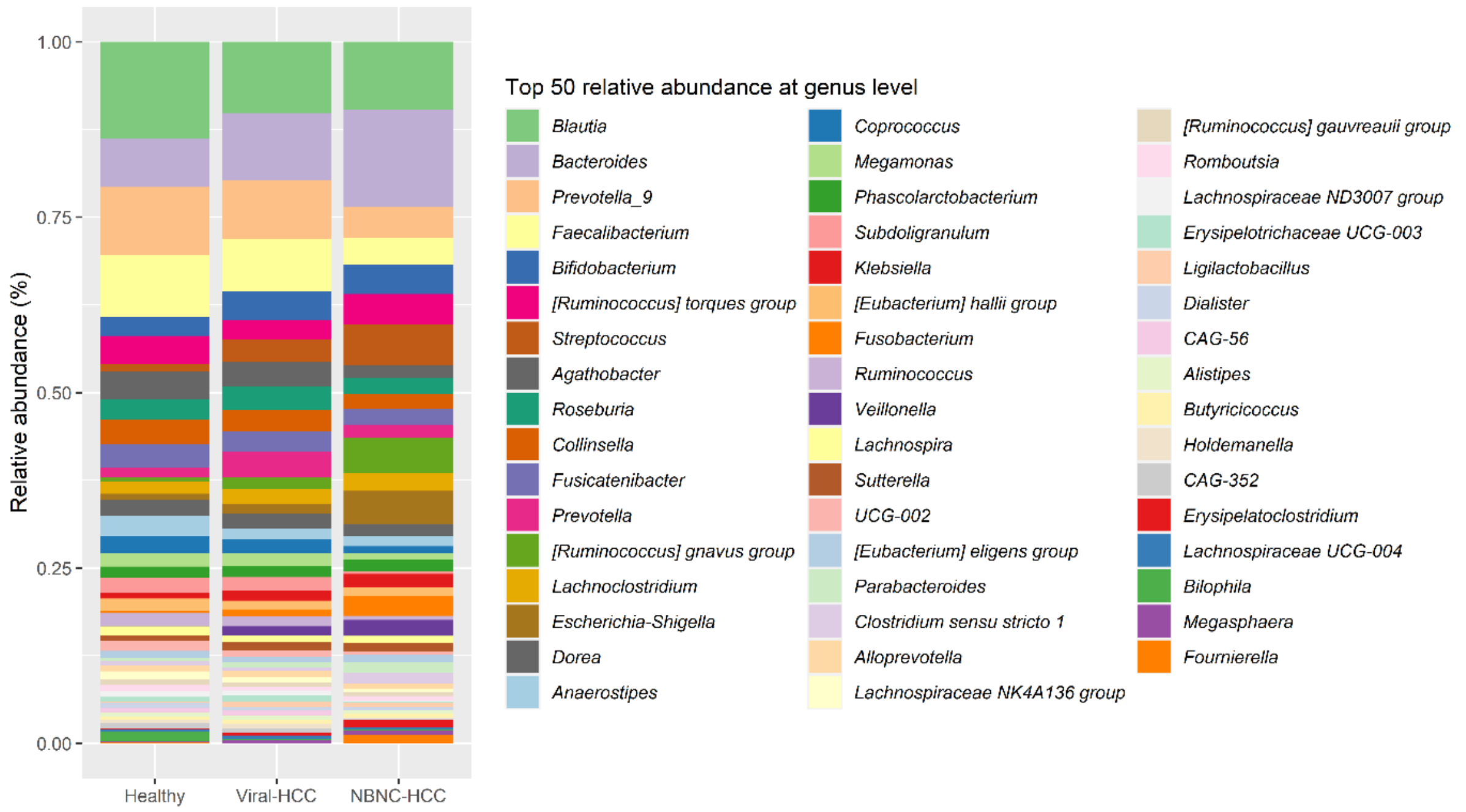

Fig. S2 Top 50 relative abundance at the genus level in healthy, Viral-HCC and NBNC-HCC
